# Sustaining a career in general practice: a qualitative study of experienced GPs in South West England

**DOI:** 10.3399/BJGP.2025.0674

**Published:** 2026-06-16

**Authors:** Nada F Khan, Bethan M Treadgold, John Campbell

**Affiliations:** 1 Exeter Collaboration for Academic Primary Care, Health and Community Sciences, University of Exeter, Exeter, UK

**Keywords:** continuity of patient care, general practice, qualitative research, service organisation

## Abstract

**Background:**

General practice in the UK faces a growing retention crisis, with many experienced GPs reducing hours or leaving patient care amid rising workload and system strain. Understanding what sustains GP careers is crucial to stabilising the workforce.

**Aim:**

To explore how experienced GPs’ career intentions have evolved since the 2016 ReGROUP study and to identify factors influencing decisions to stay, reduce, or leave clinical work.

**Design and setting:**

A qualitative interview study was undertaken with experienced GPs in South West England, and analysed using the Job Demands–Resources (JD–R) model.

**Method:**

We conducted 19 semi-structured interviews with GPs who had previously completed the ReGROUP survey. Transcripts were analysed thematically with input from public and professional stakeholders.

**Results:**

GPs described escalating clinical and administrative workloads, moral distress, and reduced control as key pressures driving burnout and exit. Hybrid access models, secondary care workload transfer, and punitive regulatory processes intensified strain. Retention was supported by clear workload boundaries, continuity of care, supportive practice systems, portfolio careers, and collective advocacy. Some GPs maintained engagement by reshaping their roles or practice structures to regain control and sustain meaning.

**Conclusion:**

Retention depends not only on reducing workload but also on restoring the conditions that make being a ‘good GP’ feel possible, through continuity, manageable demand, and supportive organisational culture. Creating space for flexibility, autonomy, and role redesign may help sustain experienced GPs and strengthen workforce resilience.

## How this fits in

Retention of GPs is critical to the stability and quality of primary care, yet many continue to leave despite policy efforts to expand the workforce. This study builds on previous work by exploring how experienced GPs navigate today’s post-pandemic general practice environment. It shows how intensifying clinical complexity, growing administrative load, and increasing accountability pressures interact to make the role feel unmanageable. Using the Job Demands–Resources (JD–R) framework, the study highlights how supportive systems, continuity, and opportunities for role redesign can buffer strain and sustain professional meaning. These insights point to practical changes at both practice and policy levels to help retain experienced GPs.

## Introduction

General practice is facing a sustained workforce crisis, with increasing numbers of GPs intending to leave direct patient care even as clinical workload and consultation rates increase.^1,2^ Recruitment has failed to keep pace with losses, leading to falling GP full-time equivalents per capita and escalating pressure on those who remain.^3,4^ Retaining experienced GPs is particularly critical because they carry the expertise needed to manage complex care, supervise trainees, support the wider multidisciplinary team, and lead practices to meet the increasingly complex demands facing primary care in the UK.^5,6^

A previous project, the ReGROUP study, explored the reasons why GPs leave practice, and identified factors including work-related stress, work–life balance, workload concerns, and a changing work environment that influenced GPs’ career decisions.^6^ Since the original ReGROUP study, general practice has faced several changes. These include shifts in service delivery during and after the COVID-19 pandemic, an increasing move to digital consultations and remote triage, and the expansion of multidisciplinary roles through the Additional Roles Reimbursement Scheme (ARRS).^7–9^

This follow-up qualitative study sought to explore whether earlier stated career intentions were borne out, and to provide deeper insights into the factors influencing experienced GPs’ decisions to stay, reduce hours, or leave. Although most participating GPs were still working in direct patient care at the time of interview, many described reducing sessions, actively reshaping their roles, or holding contingency plans to leave if conditions worsened. The study therefore focuses not only on exit, but also on what enables some GPs to continue working under increasingly difficult conditions, and where these strategies begin to fail.

We used the Job Demands–Resources (JD–R) model to inform this work. The JD–R framework distinguishes between aspects of work that generate strain (job demands) and those that support motivation, engagement, and sustainability (job resources).^10^ It is widely used to examine how demanding work environments affect retention and wellbeing. It provided a useful lens for exploring how experienced GPs balance workload pressures with resources, supports, and adaptive strategies over time.

## Method

We conducted a qualitative interview study using one-to-one semi-structured interviews between December 2024 and July 2025 in South West England.

### Sampling and recruitment

We invited GPs who had previously completed the ReGROUP GP survey in 2016, which was sent to all GPs registered to practise in South West England. The original survey response rate was 67%, and had asked about career intentions, and whether participating GPs were willing to be re-contacted regarding future research.^6^ In the present study, we approached those GPs who had originally agreed to take part in any further research.

From the subgroup of GPs willing to be re-contacted, we used purposive, iterative sampling to capture variation in age, gender, and length of time in practice. We contacted potential participants in batches of 8–10 and conducted interviews and analysis concurrently so that emerging findings (that is, differences by role type and career intention) shaped subsequent sampling. We invited 83 GPs, of whom 19 agreed to an interview. We applied the concept of information power rather than conventional data saturation to guide sample size decisions, considering our study aims and the richness of interview content.^10^ Recruitment ended when the dataset was adequate in terms of both theme development and information power.^11,12^

### Theoretical framework

We adopted a critical realist ontology and a constructivist epistemology, recognising that participants’ accounts were contextually situated while aiming to theorise about underlying mechanisms. We used the JD–R model, a unifying job design theory integrating various job stress and motivational perspectives to develop the interview guide and as a lens for grouping themes during analysis.^13^

Within this framework, we conceptualised underlying structures shaping GPs’ working lives as including material and organisational conditions such as workload volume, staffing levels, funding arrangements, commissioning rules, regulatory processes, and the organisation of multidisciplinary teams. The JD–R model supported analysis of how these structural conditions generate job demands and resources that shape GPs’ experiences, agency, and career decisions.

### Data collection

We developed a semi-structured interview guide based on existing literature, previous ReGROUP findings, and the JD–R framework. We used the guide flexibly, allowing participants to shape the conversation. The first author conducted all interviews either face to face (*n* = 2) or online (*n* = 17) depending on participant preference. Interviews lasted between 35 minutes and 62 minutes, and were audio-recorded and transcribed using Microsoft Teams or Word. The first author checked all transcripts against the audio-recordings to ensure accuracy.

### Analysis

The first author read and re-read all transcripts before coding them thematically in NVivo (version 15) using descriptive codes. The second author independently coded three transcripts, and we compared coding approaches to explore similarities and differences. We used the One Sheet of Paper (OSOP) method to organise and synthesise the data.^14^ After coding transcripts, we developed seven OSOPs across the dataset, each representing a distinct sub-theme: job demands, job strain, job resources, personal resources, proactive behaviour and job crafting, system factors outside GP control, and wanting to be a better GP. We compared these OSOPs side by side, identified overlapping themes and grouped related sub-themes into overarching themes to build the narrative results. We re-checked the narrative summary against the original sub-themes to ensure it reflected participants’ accounts and included diverse, illustrative extracts.

### Patient and public involvement and engagement and stakeholder involvement

We sought external perspectives through patient and public involvement and engagement (PPIE) and professional stakeholder input to support credibility and sense-check interpretation. A PPIE group of three members of the public reviewed the interview guide independently and suggested additional questions during a study public involvement and engagement group meeting, on GP salaries, burnout, workplace bullying, family commitments, patient complexity, and continuity of care. After interviews were completed, we shared interview excerpts with the group and held a workshop exploring patient perspectives on the data. Their insights on system pressures, GP wellbeing, work–life balance, and job sustainability informed both theme refinement and interpretation.

We took the results of this study to two GP stakeholders from ethnic minority backgrounds. Their perspectives were incorporated into the results and discussion sections.

### Reflexive notes

The lead researcher, a practising GP, brought an insider perspective that facilitated rapport with participants while remaining aware of potential assumptions. Reflexive discussions with the second author (a primary healthcare services researcher) and the PPI group alongside the GP stakeholders helped to ensure multiple interpretations were considered.

## Results

Basic demographic details of the 19 participating GPs are included in Table 1. All responders identified as being of ‘White’ ethnicity and completed their primary medical qualification in the UK or the Republic of Ireland.

**Table 1.** Participant characteristics

Participant ID	Gender	Age in years	Year qualifying as a GP	How likely permanently to leave direct patient care in the next 2 years?^a^	How likely permanently to leave direct patient care in the next 5 years?^a^	Current job role
GP 1	Male	45–50	2002	Very unlikely	Very unlikely	GP partner, 8 sessions
GP 2	Male	35–40	2003	Unlikely	Unlikely	Locum GP, 4–6 sessions
GP 3	Female	35–40	2007	Very unlikely	Very unlikely	GP abroad, 4 days a week
GP 4	Female	40–45	2000	Unlikely	Likely	GP partner, 7 sessions
GP 5	Female	40–45	2003	Very unlikely	Very unlikely	Partner, 3 sessions
GP 6	Female	55–60	1986	Very unlikely	Unlikely	Retired
GP 7	Male	50–55	1992	Very unlikely	Very unlikely	Semi-retired, 5 sessions
GP 8	Male	45–50	1996	Very unlikely	Very unlikely	Retired
GP 9	Male	50–55	1995	Very unlikely	Very unlikely	GP partner, semi-retired, 5 sessions
GP 10	Male	45–50	1998	Very unlikely	Very unlikely	Retired
GP 11	Female	45–50	1996	Very unlikely	Unlikely	Semi-retired, no sessions
GP 12	Female	40–45	2003	Unlikely	Unlikely	GP partner, 4 sessions
GP 13	Male	45–50	1998	Very unlikely	Unlikely	Academic GP, 1 clinical session
GP 14	Female	40–45	2007	Very unlikely	Very unlikely	GP partner, 5 sessions
GP 15	Female	40–45	2008	Very unlikely	Very unlikely	GP partner and locum, 5 sessions
GP 16	Male	55–60	1998	Unlikely	Unlikely	Long-term locum, 6 sessions
GP 17	Male	40–45	2009	Very likely	Very likely	Locum GP, 1–2 sessions
GP 18	Male	30–35	2014	Very unlikely	Unlikely	Salaried GP, 4 sessions
GP 19	Male	60–65	1984	Likely	Very likely	Retired

^a^These represent responses from the original ReGROUP GP survey.

Participants described their current experience of general practice as a career of contrasts, with a sense of commitment to their patients set against rising workloads, moral distress, and an increasingly fragmented and broken healthcare system. Across the interviews, five overarching themes captured how these experiences interacted to shape decisions about continuing to work as a GP, reducing hours, or leaving. A thematic map presents these results visually (Figure 1).

**Figure 1.**
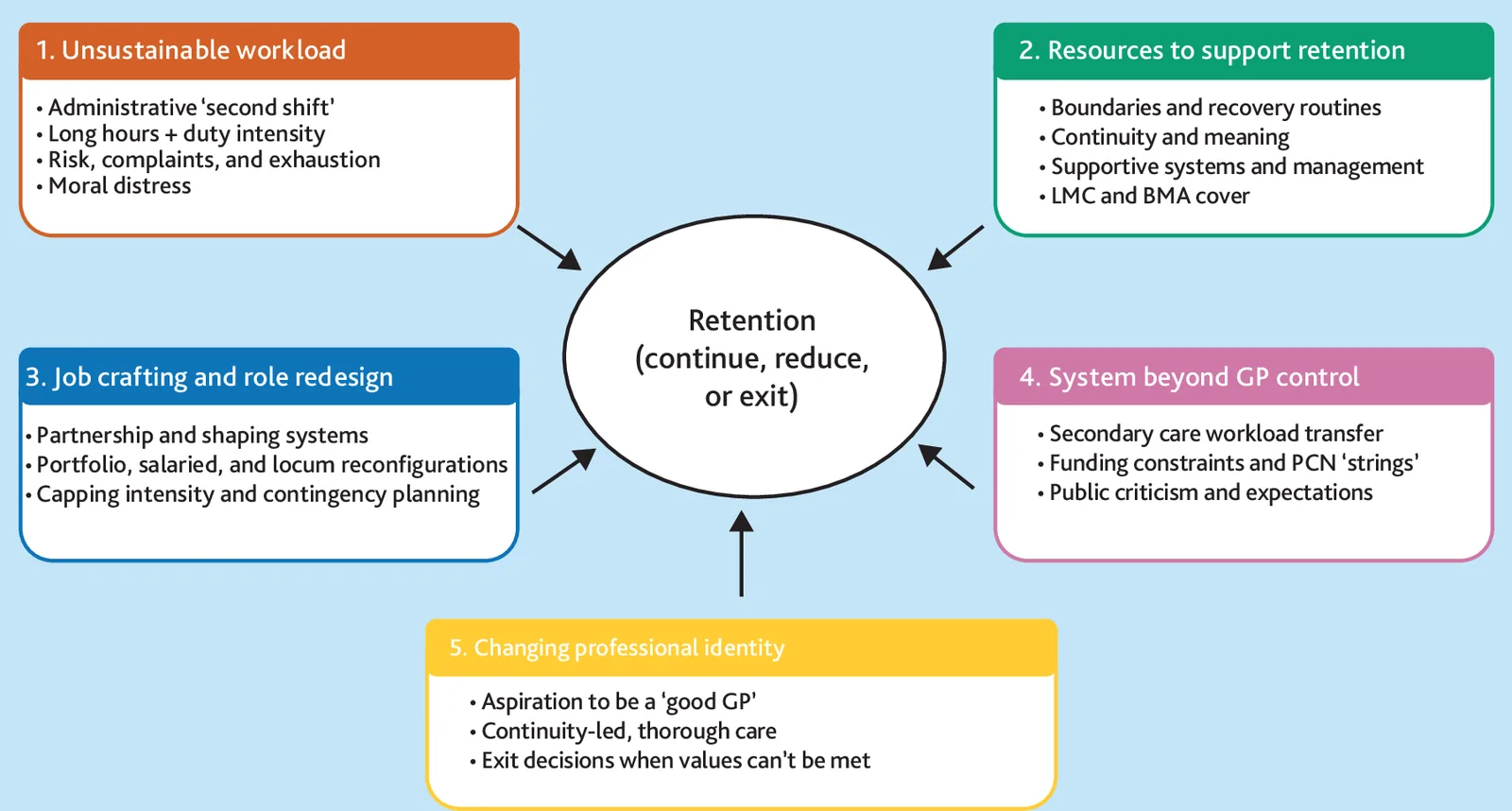
Thematic map of results. BMA = British Medical Association. LMC = Local Medical Committee. PCN = primary care network.

### Unsustainable workload: job demands and job strain

Participants described how the workload of general practice had grown in both volume and intensity, creating what many called an *‘unsustainable’* role. Rising job demands, including a greater administrative burden, long hours, clinical intensity, and moral distress, combined with job strain from increasing risk, complaints, and emotional exhaustion to push work beyond safe or sustainable limits.

#### Hidden workload and hours

Many of the GPs highlighted how administrative tasks such as managing test results, hospital letters, Care Quality Commission (CQC) work, and internal messaging including tasks were ‘unseen’ on clinical systems but had steadily expanded, spilling into breaks, evenings, and home time:

*‘But it’s quite a topic of how much admin there is to do. With lots of, you know, QOF* [Quality Outcomes Framework] *staff and CQC stuff as well* [as] *audit work. And a lot of it seems to be quite invisible. You don’t see it on your timetable … and tasks and what often happens is when your appointments saturate, you look at your task list and there’s actually seven or eight patients who’ve you’ve got a task about. It’s almost like a mini-clinic at the end of what you’re doing, which they haven’t found a space for them in the normal slots.’* (GP 1)

GPs often felt this load disproportionately against newer members of the multidisciplinary team employed through the ARRS who were, as one of our GP stakeholders noted, more protocol-driven and not expected to take on the administrative load. Our stakeholders also noted that GPs from a diverse or ethnic minority background were more likely to be in a junior role and this may contribute towards a heavier administrative load. For some, this administrative creep directly contributed to burnout and exit.

Rising demand combined with administrative overload to lengthen working days beyond formal sessions. GPs described arriving early, working through breaks, and staying late into the evening, and working up to *‘100 hour weeks’* (GP 3).

#### Clinical intensity, moral distress, and risk

GPs described clinical intensity not only as more work, but also as increasingly cognitively demanding consultations shaped by rising multimorbidity, mental health needs, and social complexity. Duty days epitomised this intensity, leaving some GPs planning retirement or switching to locum work specifically to avoid them:

*‘The issue was … the sheer volume of duty call days, where there was no limit and it would just be relentless and I felt unsafe. I felt it was poor medicine. It was a lot of batting off things that weren’t being sorted properly for the next day or the day after, and again I felt really, I felt really unhappy about that way of practising.’* (GP 6)

Digital access systems, such as eConsult or askmyGP, brought convenience for patients but also fragmentation of care from a clinical perspective, creating multiple mini-consultations and message threads that left GPs feeling overwhelmed. Several reported leaving work feeling emotionally depleted and physically exhausted with a sense of decision fatigue:

*‘I remember having* [...] *80 patient contacts on one duty day and … I said, I think I just got a lot older today and that day shortened my life.’* (GP 2)

Several linked time pressure to moral distress, or a sense of discomfort when providing care they felt was inadequate because of system constraints:

*‘I felt the issue really wasn’t about, it’s just the amount of what it was about, the quality of work. It was about, just not feeling able to do the job properly. That was my sort of moral distress.’* (GP 6)

Risk responsibility was another key stressor, amplified by remote consulting and rising referral thresholds:

*‘I'm worried that in a very short conversation there may be hints about pathology that I don’t pick up on and I’d be blamed in 6 months’ time when someone’s I don’t know, rectal cancer was missed. And of course, that’s even more risky, being a remote GP.’* (GP 17)

Complaints often tipped strain into exit decisions, with regulatory processes described as punitive rather than supportive. One GP described the emotional distress associated with preparing a response to a complaint, and feeling *‘wholly unsupported by the NHS and poorly supported by our legal counsels and legal organisations’* (GP 5)*.*

Our GP stakeholders suggested that GPs from an ethnic minority background may disproportionately face higher levels of complaints, which could impact on exit decisions.

#### From cumulative strain to tipping points

This subsection draws together the preceding accounts to show how multiple demands accumulated over time, rather than any single pressure, to create tipping points where continuing in the role no longer felt sustainable. Across the interviews, participants emphasised it was rarely a single factor but the cumulative weight and interactions of many demands, including administrative overload, long hours, clinical risk, and moral distress, which made the job feel unsustainable and contributed to a lack of control. Our GP stakeholders noted that the systems in which GPs work have taken away a sense of autonomy and control. One of the participants noted that:

*‘So I did have, have sort of a period of time where I was, I felt that I was close to burnout and I ended up sort of seeking help for that. And although it wasn’t diagnosed as burnout, I did end up having successions of psychotherapy just to see and things, and that helped … But what I, what I remember was the fact that there was sort of increasing frustration and a sense of a lack of control.’* (GP 18)

For some, the only option was reducing sessions, moving to locum or portfolio roles, or leaving altogether:

*‘Personally I would find it hard to keep, you know, keep going at that level of intensity for more sessions a week. One of the reasons I stopped doing whole long, 12-hour days because it was just too much. My brain was completely fried by the end of it.’* (GP 5)

### Resources to support retention

Alongside accounts of mounting workload and strain, participants described a set of resources and supportive mechanisms that helped keep experienced GPs in practice. These included setting boundaries around workload, managing continuity of care, establishing good practice systems, utilising portfolio careers, and adopting collective advocacy. These factors could act as a buffer against burnout, restored a sense of control, and reconnected clinicians with a sense of meaning when working as a GP.

#### Setting boundaries

Participants used the term ‘boundaries’ broadly, referring not only to limits on workload and access, but also to practices that protected time, identity, and recovery, including portfolio work and activities outside medicine. Many GPs stressed the importance of deliberately limiting workload intensity, access pathways, and out-of-hours expectations. The pandemic was described as a turning point, accelerating changes to appointment systems and access:

*‘It felt like we were at the breaking point just before COVID hit. Then obviously all that happened, but it as well as being, you know, catastrophic and deeply destructive to the way the surgery ran and we had to piece it all back together again afterwards, it did give us the opportunity to change how we worked … Phone consultations became acceptable to patients. And we felt able to start limiting our clinical work … patient expectations changed and the world changed … so you know, we have been, you know, gradually in iterative cycles changing the way that the surgery works.’* (GP 5)

Some boundaries were organisational, such as redesigning triage or controlling duty rotas, while others were personal, such as avoiding the most stressful duty shifts to avoid anxiety (GP 11). These boundary-setting strategies often overlapped with later-described job crafting practices, but were described here primarily as protective resources rather than role redesign.

For some, portfolio working combining clinical work with education or research also buffered against burnout by providing variety and preserving professional identity. One GP who had non-clinical sessions focusing on education and research described how *‘that’s what keeps me, I think in general practice is having not just patient facing aspects to my work’* (GP 15).

Another GP who worked in a number of senior roles in the NHS described that *‘the interesting things about all that it created variety in in the job I was doing, so I wasn’t just hammered by the day-to-day grind of individuals coming to see their GP’* (GP 19).

Many GPs spoke about deliberately cultivating recovery routines to keep the job sustainable. This often involved exercise, being in nature, or creative activities such as music and gardening. These routines outside of work could foster a sense of resilience.

#### Continuity and professional meaning

Continuity of care was repeatedly described as the heart of satisfying practice, being both clinically efficient and emotionally rewarding, shifting fragmented work into coherent, meaningful care:

*‘I think it’s one of the key things of the job … I think there’s no doubt that you get this additive understanding over time of people and they hopefully confide in you more or feel more comfortable to talk to you about things … so I think a lot of the satisfaction for me is in the ongoing relationship mutually.’* (GP 1)

By contrast, fragmented care generated inefficiency and frustration:

*‘If it’s someone else’s* [patient] *… it takes about 10 minutes to read and understand before I’ve even got the patient in the room.’* (GP 4)

Some GPs reflected on the diagnostic satisfaction that continuity brings, including spotting subtle changes that led to early diagnoses, describing this as the *‘what you’ve got to feed on basically, that’s the positive part of primary care’* (GP 19).

Others described gratitude and shared history with families and communities as central to why they stayed, as this GP describes how *‘that’s really important and that that’s probably one of the reasons why I haven’t left to go anywhere else’* (GP 15).

#### System design and collective advocacy

Practices with well-designed appointment systems, strong management, and responsive leadership described a sense of control and sustainability often missing elsewhere. One GP described a system where the duty doctors sat alongside receptionists doing triage together as working well for clinicians and patients:

*‘*[We’ve] *got a really sophisticated triage and appointments organisation system … and so there’s this great shared decision making between the clinicians and the receptionists who really know what’s going on in the practice and all the different slots people can be put in … so everybody gets triaged, but they get triaged really effectively. And in a way that satisfies patients as well as satisfies the system. It’s amazing.’* (GP 13)

Local Medical Committee (LMC) and British Medical Association (BMA) advocacy enabled practices to push back against inappropriate workload transfer, legitimising local boundaries and reducing isolation:

*‘Now if I’m doing a piece of work and I really think there’s a very low yield to it or I can’t see the point of it, then certainly you can flag it and say why am I doing this? If it’s a recurring theme we can set, we can approach the LMC and say, hey, look, we’ve noticed a lot of this stuff is coming back to us and it seems a bit kind of below our pay grade … I think if they’re recurring problems with the same themes, we kind of notice that and then we can kind of get a more of a corporate response.’* (GP 1)

### Job crafting and role redesign

Some participants described deliberately reshaping the structure, scope, and intensity of their work through a process several called *‘job crafting’*. This ranged from tinkering with practice systems to redesigning individual roles or planning exits when change or role redesign was not supported.

#### Shaping practice systems: partners as agents of control

Practice partners in particular described actively re-engineering appointment systems, IT platforms, and staffing models to make work safer and more sustainable. This system-level control was described as a key motivator for partnership itself, allowing the ability to drive meaningful change to attenuate external pressures:

*‘I get a lot of satisfaction out of knowing that I am part of keeping a small practice and a small patch of Cornwall well served by a really, really good GP practice that’s high quality and that we’re giving the absolute best service we can with the funding and the staffing available to us. So I get, yeah, I get a lot of sense of satisfaction out of that. So that’s part of why, why I do what I do, not just the clinical work.’* (GP 5)

Examples included introducing paramedics and pharmacists into on-call teams or shifting whole primary care networks (PCNs) onto unified digital systems for efficiency and safety. This kind of system redesign created time and space for GPs to focus on complex care and continuity, although some warned that poorly aligned new multidisciplinary roles occasionally added supervision demands rather than reducing workload. Our GP stakeholders also describe the consequences of this shift of increasingly complex care to GPs making clinical sessions more intense:

*‘Some things, only GPs can manage or, they, you know, they* [Advanced Nurse Practitioners, ANPs] *may have learning needs that might, you know, it actually increases workload. It’s, yeah, things like that are a bit frustrating because the patient’s getting delays to their care. Patients had to have involvement from three clinicians rather than one if it was a GP in the first place, then maybe that would have been quite simple to sort out.’* (GP 15)

#### Redesigning roles: flexibility, variety, and boundaries

Alongside system changes, many GPs reconfigured their own roles to set boundaries. For some, partnership remained the route to autonomy and leadership, as this GP who moved from a salaried to a partnership role describes:

*‘That aspect that I can have more control* [as a partner]*, you can’t have any control over what patients are doing in terms of demand. So at least if I can have some control over how we’re working … I’m the one who’s trying to drive us to work smarter, but not shortening appointments, but working smarter in other ways, prescription management, you name it.’* (GP 12)

Others, however, stepped back from leadership responsibility, moving into salaried, portfolio, or locum roles to reduce financial and clinical risk, and regain control over time and boundaries:

*‘And with management responsibility, with the authority comes the responsibility, and sometimes it’s nice not to have that responsibility. So to some degree …’* (GP 8)

Locum work offered maximum flexibility for some, particularly around childcare and family life, although several noted that changing demand and funding had eroded this option over time:

*‘There’s been a huge change in that the, I mean the availability has gone almost to nil … and many of the locums simply have very little work. So I would say in the last 2 years, but especially in the last year, the demand for locums has almost stopped completely.’* (GP 16)

#### Exit decisions and contingency plans

Job crafting often ran alongside exit planning. Emigration or career breaks were often framed as contingency plans if intensity rose or support fell away. One GP, who emigrated to Australia, described how:

*‘Out here is very positive because it feels so flexible … I really don’t know how I would go back to the NHS as it is because I don’t want to be a partner, I don’t want all the responsibilities and locking in ... that comes with that … but as a salaried I wouldn’t want to answer to somebody else or, you know, be stuck there every evening with no say in my working hours.’* (GP 3)

For others, specific crises including complaints, illness, or threats from patients accelerated decisions to step back:

*‘Someone threatened* [me] *so severely … I went away for 2 weeks … and I was like, what is going on here? This is not right. And I came back and I handed in my notice and I became a locum. So that was how that happened.’* (GP 2)

### The system outside my control

Participants repeatedly described general practice as absorbing the failures of a fragmented and under-resourced system, a theme that expanded beyond the JD–R framework. This promoted a tension where GPs were left carrying the risk for system gaps outside of their control.

#### Fragmented systems and workload transfer

Participants described how delayed discharges, under-resourced community services, and growing secondary care waiting times all spilled over into general practice. GPs found themselves managing the subsequent consequences without the capacity or resources to fix the upstream problems. Secondary care *‘workload dumping’* including rejected referrals, *‘ping-pong’* advice and guidance, and requests for GP monitoring of specialist pathways left GPs holding additional risk:

*‘And so in particular, the sort of advice and guidance system, it’s a great idea … whereas in actuality what’s happened is it becomes a bit of a ping-pong match. Where we’re asking for help, the secondary care team … sort of bounce it back and then for various reasons we’ve bounced it back saying we’ve tried it, or we can’t provide that level of care, and then they’ll come up with another solution and bounce it back. And this is over a period of weeks or months. It means the GPs … are carrying a considerable clinical risk that something will go wrong.’* (GP 16)

These gaps in care pathways left GPs feeling that they were constantly *‘patching up’* system failings while bearing the residual medicolegal risk:

*‘It feels like we’re trying to help people navigate a system that is broken while our own system’s a bit broken too.’* (GP 17)

Narrowly commissioned services left patients *‘bouncing’* between providers when patients fell outside rigid criteria, such as right-sided heart failure or complex mental health needs:

*‘The community services like heart failure nurses, you know, I realise that their remit was so narrow they would only manage left heart failure. And I had a patient who was really struggling with massive right heart failure, doesn’t tick a box, doesn’t follow that. So the fact* [is] *that we were not quite sure what to do with him in the end because things were getting so difficult.’* (GP 6)

GPs described this way of working as clinically unsafe and emotionally exhausting.

#### Rising demand and changing expectations

Post-pandemic, GPs felt patient expectations had shifted dramatically, with an increasing demand for faster access and more direct digital contact alongside extra tasks generated by private care:

*‘People just sort of drop in an eConsult asking me to write a letter and stuff like this, you know, I did sort of secretarial work … It doesn’t feel* [like a] *hugely rewarding thing to do. You know, I’m not sure I needed all my training to write a letter to a private consultant.’* (GP 14)

Our GP stakeholders described the increasing burden of right to choose referral processes adding to workload. In this study, some GPs linked this rising demand to declining continuity, fuelling inefficiency and less time for more complex patients:

*‘We’ve drifted into a system of where everything is same-day care … it’s commercialisation of general practice, sort of like seeing it like a 24-hour supermarket in terms of things or I should have access all the time, I should be able to access anywhere I want. And I think that’s the thing … we’ve gone to that model of care, and that means that sort o*f *we’re trying to manage all of those responses, but that* [they] *don’t need to actually be the same day, but we’re still managing them same day. It means that we’re sort of harder pushed to sort of like actually sort of see effectively the people that are bit more complex and need that sort of time in terms of things.’* (GP 18)

#### Financial constraints and organisational friction

Despite rising workload, participants described an environment of decreasing, complex, and unpredictable funding. Partners reported struggling to fund estate upgrades, meet rising salary expectations, or flexibly use PCN funding tied to restrictive rules:

*‘As time went by I realised that the PCN was a load of nonsense … we were being tied in knots by sort of working out how we were going to get funding. And if we didn’t spend the money, we wouldn’t get the money. And if they* [had] *just given us the money and let us do our job, you know, so you can employ who you want, then it would have been great. There’s always so many strings attached.’* (GP 2)

For several participants, these financial and organisational constraints tipped the balance towards reducing sessions or planning early retirement.

#### Negative narratives and professional identity

Alongside these structural pressures, GPs felt publicly blamed for system failings, with negative media rhetoric and adversarial complaint processes eroding morale and professional identity:

*‘Make the job something attractive. Stop hammering GPs. Stop the* Daily Mail *attacking primary care all the time. For goodness’ sake, that’s people who just don’t understand the social service, if you like, we provide just by looking after people and keeping them out of hospital.’* (GP 19)

Professional identity, and being seen as a specialist, was important to some GPs who felt that the complexity that they were managing was not acknowledged by the public:

*‘The general public don’t see us as qualified as consultants whereas actually we are the generalist specialist … I would say we’d really need to push recognition as specialists … I think we need to be recognised as specialist really because we look after everybody all the time for everything. And I do think that that it’s quite morale breaking for people to sort of view us as they do and yet we are doing really complex work.’ (*GP 12)

Such experiences fuelled a strong sense of being undervalued compared with hospital consultants, despite carrying wide-ranging clinical responsibility.

#### Emotional consequences and exit decisions

For some, the cumulative impact of a low-control, high-responsibility role was sadness at the state of their general practice career, and a feeling that working as a GP no longer aligned with the kind of care they wanted to provide. Some described staying only by reducing clinical hours; others retired earlier than planned or emigrated to work in better-resourced settings. Our GP stakeholders felt higher salaries would help, but as one of our responders put it:

*‘I fundamentally think that like no money in the world will keep people doing a job that they actually actively hate.’* (GP 18)

This sense of sadness could lead to action including reducing clinical sessions, retiring earlier than planned, or moving abroad to work in systems perceived as better resourced and better aligned with the kind of medicine they wanted to practise.

### Changing professional identity: wanting to work better

Finally, participants reflected on what it means to be a ‘good GP’ amid these changing conditions. Many contrasted earlier careers with the current duty-heavy, remote, and time-pressured models. This shift was particularly evident when working with older, frail patients and those with complex multimorbidity. Many contrasted the intrinsic satisfaction of home visits, palliative care, or care-home rounds with the demoralising and intense work associated with duty days.

Some recalibrated expectations, shifting from a *‘platinum service with a cherry on top’* to *‘a safe silver service’* within available time (GP 18). Others felt that only very low clinical volume allowed them to deliver care aligned with their values, as this GP who reduced his clinical sessions to manage the workload describes:

*‘And I could practise quicker. I could do shorter consultations. I could write less helpful referral letters. I could not go the extra mile for people and still do adequate general practice and probably get out at 13:30. But I just can’t … You know, one of the pleasures of the job is, you know, sorting people out and doing things well.’ (*GP 13)

#### Seeking alternative ways to be a doctor

Some participants sought roles that restored time and control including moving overseas, shifting to military medicine, or considering private GP work. Emigrating was described by a few of the responders as a way to practise medicine how they wanted to and to protect their own boundaries:

*‘I can’t carry on in this job as I’m doing it now, and therefore this seems like the last window to go to Australia.’* (GP 3)

Private GP work was often framed reluctantly, as a last resort for reclaiming time, autonomy, and quality:

*‘I could see myself doing private GP and I hate to say that I really hate to say that, but the reason I could is because that the, you know, the more time, the more, less pressure … and so having a bit more freedom to do the right thing for the person would make me feel a lot, give me much more job satisfaction, much more feeling that I was doing the right thing. It’s the constraints of the current model that feel risky and at odds with my beliefs, my values.’* (GP 17)

Across the interviews, the aspiration to be the ‘good GP’ through continuity-based thorough general practice was clear. Where systems enabled boundaries, team support, and realistic scope, clinicians often found ways to stay in practice. Where the model made ‘good GP’ work feel impossible, they crafted their role downward, or stepped away from NHS general practice entirely. This reflected a growing mismatch between professional standards and the conditions under which those standards could be met.

## Discussion

### Summary

This in-depth qualitative study explored the experiences of GPs in the South West of England, focusing on factors influencing retention and exit decisions. Participants described a career of contrasts, balancing a commitment to patients against rising workloads, moral distress, administrative burden, clinical risk, and a fragmented healthcare system.

Using the JD–R model as a lens, we identified five overarching themes shaping experienced GPs’ decisions to stay, reduce or recraft their work, or leave. Unsustainable workload reflected the job demands side of the model, with administrative overload, long hours, clinical intensity, emotional exhaustion, and risk responsibility driving strain and burnout. Resources to support retention represented job resources, where boundary-setting, continuity, portfolio careers, supportive systems, and collective advocacy buffered workload and restored meaning. Job crafting and role redesign aligned with the JD–R in describing proactive efforts to reshape work demands and resources to sustain engagement. System pressures beyond GP control showed how external constraints, including workload transfer, funding limits, and public criticism, intensified demands and reduced professional control. Finally, changing professional identity highlighted the motivational dimension of the JD–R model, where rising demands and reduced resources undermined professional values, prompting some GPs to consider leaving their role.

These findings demonstrate how escalating pressures, insufficient support, and reduced autonomy threaten the sustainability of experienced GP careers, while restoring boundaries, control, and professional meaning could help create the conditions needed to retain experienced GPs in the workforce.

### Strengths and limitations

This study used iterative, purposive sampling to capture a range of experiences across career stages, genders, and working patterns. PPIE input shaped both data collection and we included GP perspectives when shaping the analysis and interpretation. Limitations include the regional focus on South West England and self-selection bias, as participants willing to be interviewed may have held stronger views about workload or retention. In terms of the sample, we only spoke to GPs who identified as being of White ethnicity, but this reflected the sample of the original ReGROUP study, of which 93.4% of the original GP responders were of White ethnicity. GPs from other ethnic groups were approached but none agreed to an interview. We included GP stakeholders from a broader ethnic background to try to accommodate a more diverse view on the study interpretation and findings.

### Comparison with existing literature

Our findings align with and extend existing research showing that GP attrition reflects the cumulative impact of administrative overload, rising clinical complexity, and increasing perceived risk.^5,15,16^ Consistent with other research, we found that declining continuity and expanding non-clinical workload erode job satisfaction and professional identity.^17,18^

This study adds to the literature in three ways. First, in the post-COVID context, it shows how hybrid access models (eConsult and telephone triage), escalating duty day intensity, and advice-and-guidance *‘ping-pong’* have reshaped daily work, creating fragmented micro-consultations, stacked asynchronous tasks, and additional cognitive load from displaced secondary care follow-up. Second, we extend the job resources side of the JD–R model by detailing how bounded duty lists, protected administrative time, continuity pathways, practice management capacity, and LMC and BMA advocacy can restore control and meaning, aligning with but building on prior research on GP autonomy, teamwork, and continuity as protective.^5^ Third, we highlight job crafting as a pivotal response. GP partners in particular used system redesign to reclaim control, while others developed portfolio or salaried roles to cap workload intensity, echoing occupational psychology research on job crafting.^19,20^

Finally, we highlight the values GPs brought to their role. Many GPs felt morally compromised when unable to deliver the continuity and quality of care they considered central to good practice. For some, this was the tipping point towards reducing sessions or leaving altogether. While moral strain among clinicians has been described before, our findings show how, for GPs, it combines with workload pressures to create a low-control, high-responsibility environment that can drive career decisions.^21^ Retention therefore relies not only on reducing workload but also on restoring the conditions that make being a ‘good GP’ feel achievable through continuity, manageable demand, and practical organisational support.

### Implications for research and practice

Our findings highlight actions for the practice, system, and policy levels (see Box 1 for a summary).

**Box 1.** GP retention practice- and system-level strategies

Practice-level strategies	System and policy-level strategies
*Set and protect workload boundaries* Follow BMA recommendations to limit duty lists, cap appointment volumes, and build protected administrative task time	*Commissioning arrangements* Prevent inappropriate transfer of prescribing, follow-up care, and administrative tasks from hospitals to GPs
*Redesign practice systems* Implement efficient triage models to redistribute clinical and administrative work within the MDT	*Complaints and regulation reform* Develop fair, timely, and supportive complaints investigations with access to pastoral care
*Protect continuity of care* Allocate appointments to the same GP for episodic or relational continuity to restore professional satisfaction	*Sustainable funding* Invest in core general practice funding rather than relying on short-term or ring-fenced schemes for recruitment, estate upgrades, and supervision time
*Support flexibility and portfolio careers* Provide sessional flexibility, job-sharing, or portfolio careers incorporating teaching, research, or leadership	*Retention metrics* Incorporate retention metrics into workforce planning to track actual GP exits rather than relying on intentions to leave

BMA = British Medical Association. MDT = multidisciplinary team.

At the practice level, setting and protecting workload boundaries is essential through following suggestions, such as those from the BMA workload recommendations to limit duty doctor lists, cap appointment volumes, and build protected administrative time into the working day.^22^ Redesigning practice systems is challenging, but can increase patient satisfaction and manage workload through efficient triage models to redistribute appropriate clinical and administrative work within the practice multidisciplinary team (MDT).^23^ Protecting continuity of care is another priority. Redesigning systems to allocate appointments to the same GP either for episodic or relational continuity can help restore the professional satisfaction that comes from knowing patients well.^24^ Similarly, supporting sessional flexibility, job-sharing, or portfolio careers that incorporate teaching, research, or leadership opportunities can help retain experienced GPs who might otherwise leave owing to workload intensity or lack of control over working patterns.^25^

However, practice-level strategies can only partially mitigate against wider pressures. At the system level, commissioning arrangements should avoid unresourced transfer of prescribing responsibilities, follow-up care, or administrative tasks from secondary care onto GPs without capacity or funding. Reforming complaints and regulatory processes is also a priority. Participants in this study repeatedly described the current approach as punitive and demoralising, suggesting the need for timely, fair, and supportive investigations with access to pastoral care for clinicians. Echoing the Royal College of General Practitioners (RCGP) and BMA, investment in retention schemes should be available for those at risk of leaving the profession.^25^ Finally, workforce planning should move beyond recruitment targets to incorporate retention metrics that track actual patterns of experienced GPs leaving clinical work, rather than relying solely on intentions to leave.^25^

Future research should prioritise testing practical interventions to improve GP retention. Evaluation of redesigned triage and duty systems, administrative task sharing, or continuity pathways could clarify which organisational changes most effectively reduce workload and prevent burnout. Research should also explore whether experiences of workload intensity, moral distress, ability to ‘job craft’, and drivers of attrition and retention differ by ethnicity or gender. This is important to ensure future policies address the needs of a diverse workforce, especially with increasing numbers of international medical graduates working in UK general practice.^26^

In conclusion, experienced GPs described both the pressures pushing them out of the profession and the resources that help them stay. Addressing professional workload, autonomy, and identity is essential not only to retain experienced GPs but also to protect continuity, quality, and sustainability in primary care.
